# Dogs’ reaction to inequity is affected by inhibitory control

**DOI:** 10.1038/s41598-017-16087-w

**Published:** 2017-11-17

**Authors:** Désirée Brucks, Friederike Range, Sarah Marshall-Pescini

**Affiliations:** Comparative Cognition Unit, Messerli Research Institute, University of Veterinary Medicine Vienna, Medical University Vienna, University of Vienna, Vienna, Austria

## Abstract

Inequity aversion is thought to act as a mechanism to ensure cooperation and has been studied in many different species, consistently revealing inter-individual variation. Inhibitory control has been proposed to act as one factor responsible for this variation since individuals need to inhibit performing the required action and/or refuse rewards in order to exhibit inequity aversion. Here, we investigated if dogs’ sensitivity to inequity is affected by their capacity for inhibitory control, assessed in a test battery and questionnaire. Overall, dogs showing high compulsivity scores (i.e. repetitive behaviours independent of feedback) were more motivated to participate in the inequity task independent of the rewarding scheme. Dogs were more sensitive to inequity and individual contrast if they exhibited a slower decision speed in the inhibition tasks. Furthermore, less persistent and more impulsive dogs were more sensitive to reward inequity, potentially due to having a lower tolerance level for frustration. Results indicate that aspects of inhibitory control can explain the variation in dogs’ inequity response, highlighting one of the mechanisms underlying responses to inequity. Emphasising the importance to design paradigms, which allow us to disentangle capacities to recognise inequity from the inability to react to it due to poor inhibitory control abilities.

## Introduction

Cooperation with other individuals in order to achieve a goal that could not be reached alone seems to be clearly advantageous. However, cooperative interactions are mostly beneficial if the payoff is shared more or less equally among all individuals that contributed to the action. If this is not the case, unequally treated individuals may stop their participation and look for other, potentially better, cooperative partners. Therefore one mechanism likely to stabilize cooperation is inequity aversion, the negative reaction to unequal treatment^1^. Inequity aversion seems to be present across many different animal taxa (i.e. apes (e.g. ref.^2^), monkeys (e.g. ref.^3^), corvids^4^, rodents^5^, canids^6^) indicating that this behaviour might be a basic trait shared across different social species^7^. Typically, inequity aversion has been assessed by requiring pairs of individuals to alternately perform an action (e.g. exchanging a token, giving a paw or pulling a tray) in order to receive rewards, which are either equally or unequally distributed between partners. If an individual refuses to continue participating in the task after witnessing that their partner received a reward of better quality for the same amount of work (by either stopping to perform the required action or refusing to accept the reward), it is considered to show inequity aversion.

Inequity aversion research has focused mainly on investigating the existence of this behaviour across different species (see ref.^8^ for a review) and only recently the individual variation in the reaction to unequal treatment has been given attention (see ref.^9^ for a review). Considering the nature of inequity aversion as a mechanism to stabilize cooperation, it comes as no surprise that an animal’s reaction to inequity may differ depending on the relationship with its social partner (i.e. affiliation^2,10,11^, but see refs^12,13^; dominance^5,14–16^, but see ref.^11^). But also individual characteristics, such as age^17^, sex^10,15^ (but see ref.^18^), and certain aspects of personality^12^ seem to play a role in the response to unequal treatment.

Another factor that has consistently been suggested to influence an individual’s reaction to inequity is inhibitory control (e.g. refs^19,20^). Inhibitory control is referred to as the ability to overcome the temptation of performing an immediately rewarding behaviour in favour of a delayed but more appropriate (and ultimately more rewarding) one. While social as well as some individual factors might account for the *susceptibility* to inequity, inhibitory control is always necessary for *exhibiting* an aversion to it. In order to show an aversion to inequity, an animal needs to inhibit delivering a token, pulling a tray or giving the paw, despite having been consistently rewarded for carrying out this action or refuse to take a food reward of lesser quality than the partner. Thus, the ability to inhibit cooperative interactions in unequal situations might be an important cognitive prerequisite involved in the expression of inequity aversion, and could potentially account for the wide-ranging individual variation consistently observed across studies.

To our knowledge, no study so far has investigated the relationship between inhibitory control and inequity aversion directly. However, some studies looked at other cooperative behaviours likely linked to inequity aversion in humans (e.g. altruism^21^, reciprocity^22^, sharing behaviour^20^). Of particular interest is a study by Harris and Madden^23^ that found a relation between cooperative interactions in a prisoner’s dilemma game and individual variation in inhibitory control. Accordingly, more impulsive people, as measured in a delay-discounting task, defect unequal offers more often than less impulsive people, who try to maintain offers for longer. In non-human animals, only one study investigated the link between inequity aversion and inhibitory control. Capuchins monkeys were found to exhibit better inhibition in a detour-reaching task than tamarins and authors related these findings to results from inequity aversion studies showing that while capuchins do show inequity aversion, tamarins do not react to unequal treatment^19^. However, Lakshminaryanan and Santos did not obtain measurements of inequity aversion from the same monkeys that were tested in the inhibitory control task and consequently, the authors could only make inferences about a possible relationship between these factors without having the actual data from both inequity and inhibition task to test this hypothesis directly.

One problem that arises in testing the involvement of inhibitory control in other behaviours is its strong context-specificity (e.g. refs^24,25^). For example, human’s inhibitory abilities cannot be easily captured with a single task, which raises concerns in regard to the above-mentioned studies (e.g. refs^20,22,23^). Similarly, in a recent study with dogs^26^, we found that inhibitory control abilities in this species also vary across tasks, and hence it cannot be considered a unified concept measurable in a single test. Nonetheless, we could show that dogs’ inhibitory control abilities, which were assessed across five inhibition tasks, are likely explained by three components: persistency (i.e. a measure of dogs’ persistence in manipulating apparatuses), compulsivity (i.e. a measure of dogs’ consistently choosing the same option despite of not being rewarded) and decision speed (i.e. the speed with which dogs made their choices).

The aim of the current study was therefore to investigate the role of inhibitory control (and more specifically its three main identified components) in the expression of inequity aversion in dogs. Dogs’ reaction to inequity was measured in a paw-giving paradigm, which has elicited inequity aversion in dogs in previous studies^6,11,27^. In this paradigm pairs of dogs were alternately asked by an experimenter to give their paw and received either the same or different reward types for performing this action^27^. In addition to testing the dogs’ reaction to unequal treatment, we also incorporated a condition specifically testing for frustration/individual contrast (i.e. showing the more preferred reward but the giving the less preferred one). Measures of inhibitory control were obtained from a test battery consisting of five frequently used inhibition tasks^26^, including a validated impulsivity questionnaire^28^. We hypothesized that inhibitory control is linked to inequity aversion, in particular predicting that dogs showing better inhibitory control abilities are more sensitive to inequity but also to individual contrast. However, considering that inhibitory control is a complex construct, which is best described by three components in dogs (persistency, compulsivity, decision speed), we expected these components to be differentially involved in the expression of inequity aversion.

## Results

### Overall motivation to work in the inequity paradigm (Model 1)

Model reduction revealed that the persistency and compulsivity components had an influence on the overall number of times the paw was given, independent of test condition. While the compulsivity component had a significant positive effect on paw giving in the whole inequity paradigm (GLMM: *b* = 0.23, *SE* = 0.06, *z* = 3.74, *p* < 0.001), the persistency component was not significant (GLMM: *b* = 0.11, *SE* = 0.07, *z* = 1.63, *p* = 0.103). Accordingly, dogs showing more compulsive behaviours in the inhibition tests gave their paw more often than dogs showing fewer of these behaviours. This indicates that dogs, which consistently stuck with their choices in the inhibition tests independent of the feedback ( = compulsivity component), reacted less strongly to the differential rewarding schemes in the inequity test.

### Relationship between inhibition and reaction to inequity and contrast in ‘dyadic conditions’ (Model 2)

**Figure 1:**
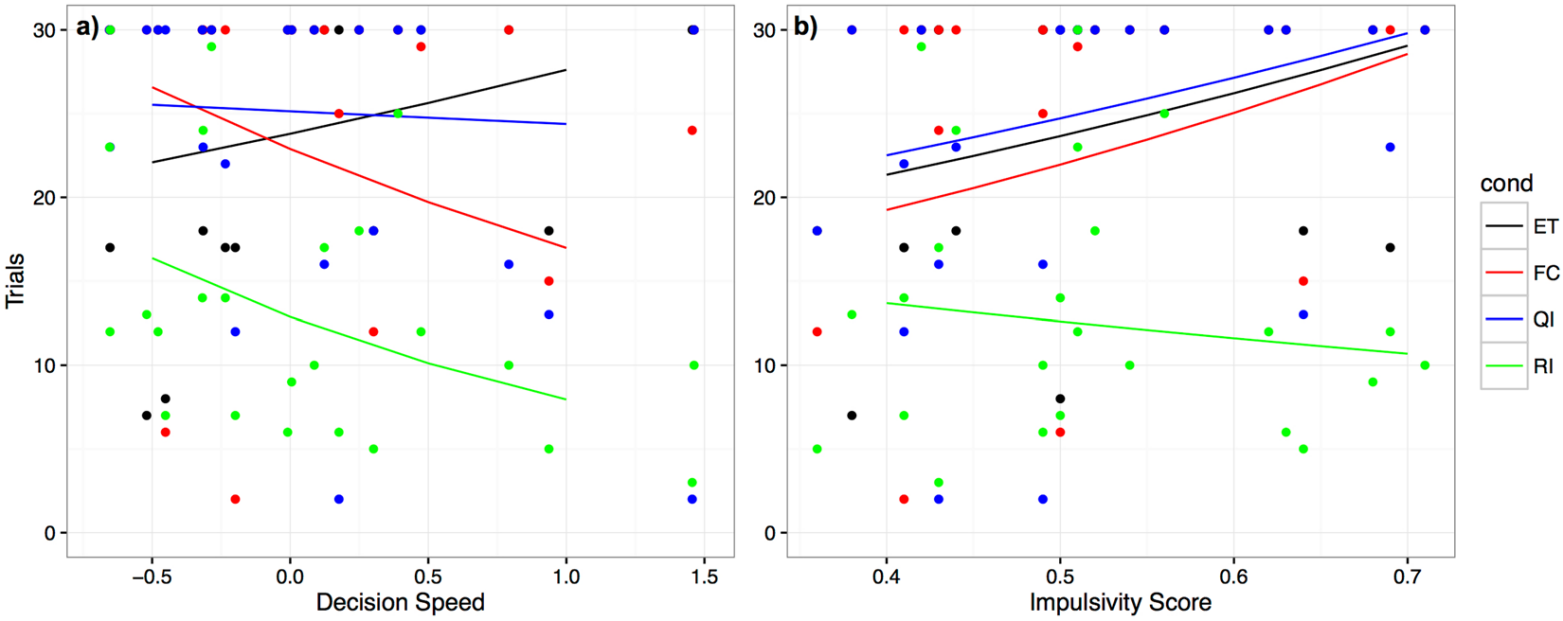
(**a**) Interaction between numbers of trials completed (= n. times paw given) per condition and the decision speed component. A higher score on the decision speed component is associated with a slower reaction in the inhibition tests. (**b**) Interaction between numbers of trials completed per condition and the impulsivity score obtained from the questionnaire. A low impulsivity score indicates that a dog is less impulsive whereas a high score means that a dog is very impulsive. Raw data (points) and predictions based on the selected model (colours: black = ET, red = FC, blue = QI, green = RI).

The reduced model for the dyadic conditions (equity condition as baseline) included the coefficients decision speed, and the impulsivity score derived from the DIAS questionnaire. Both coefficients showed significant interactions with some but not all test conditions (see Table 1). We found a significant negative interaction between the decision speed component and the food control as well as the reward inequity condition, while this interaction was only a trend for the quality inequity condition (see Table 1, Fig. 1a).

**Table 1:** Effects of inhibition components on paw-giving behaviour in dyadic conditions

Fixed effects	Estimate ± SE	*z*-value	*P*
*Food Control* (*FC*)	−0.19 ± 0.32	−0.58	0.563
*Quality Inequity* (*QI*)	0.10 ± 0.32	0.32	0.747
*Reward Inequity* (*RI*)	0.34 ± 0.38	0.89	0.375
*Decision Speed*	0.15 ± 0.11	1.34	0.174
*DIAS Impulsivity Score*	1.03 ± 0.68	1.51	0.132
FC*Decision speed	−0.45 ± 0.10	−4.41	<0.001**
QI*Decision speed	−0.18 ± 0.09	−1.90	0.057
RI*Decision speed	−0.63 ± 0.13	−4.97	<0.001**
FC*DIAS Score	0.29 ± 0.61	0.48	0.634
QI*DIAS Score	−0.09 ± 0.60	−0.16	0.877
RI*DIAS Score	−1.85 ± 0.73	−2.52	0.012*

*p < 0.05; **p < 0.01.

Consequently, dogs with a slow decision speed stopped giving their paw earlier in these three conditions (FC, QI, RI) in relation to the baseline (ET), while dogs with a faster decision time continued giving their paw for more trials. Thus dogs that took longer to make a decision in the inhibition tests also showed a stronger response to inequity, but also to contrast. Additionally, the DIAS impulsivity score, showed a significant interaction with the dogs’ paw-giving behaviour but only in the reward inequity condition (see Table 1). Accordingly, dogs with a higher impulsivity rating stopped giving their paw earlier (see Fig. 1b). More impulsive dogs showed a stronger response to reward inequity than less impulsive dogs.

### Relationship between inhibition and reaction to reward inequity in ‘unrewarded conditions’ (Model 3)

In addition, we ran a model including only the unrewarded conditions, hence considering the asocial no-reward condition as baseline. The best-fit model included all coefficients and their interaction with the test conditions (see Table 2.). Of those coefficients only the interaction with the persistency and decision speed components were significant (see Table 2.).

**Table 2:** Effects of inhibition components and DIAS impulsivity score on paw-giving behaviour in unrewarded conditions.

Fixed effects	Estimate ± SE	*z*-value	*P*
*Reward Inequity* (*RI*)	−0.55 ± 0.39	−1.38	0.166
*Persistency*	−0.03 ± 0.12	−0.26	0.794
*Compulsivity*	0.15 ± 0.11	1.43	0.152
*Decision Speed*	−0.01 ± 0.12	−0.09	0.925
*DIAS Score*	−1.30 ± 0.70	−1.87	0.061
RI*Persistency	0.27 ± 0.13	2.12	0.034*
RI*Compulsivity	0.16 ± 0.11	1.45	0.148
RI*Decision Speed	−0.49 ± 0.14	−3.40	<0.001**
RI*DIAS Score	0.11 ± 0.77	0.15	0.881

*p < 0.05; **p < 0.01.

**Figure 2:**
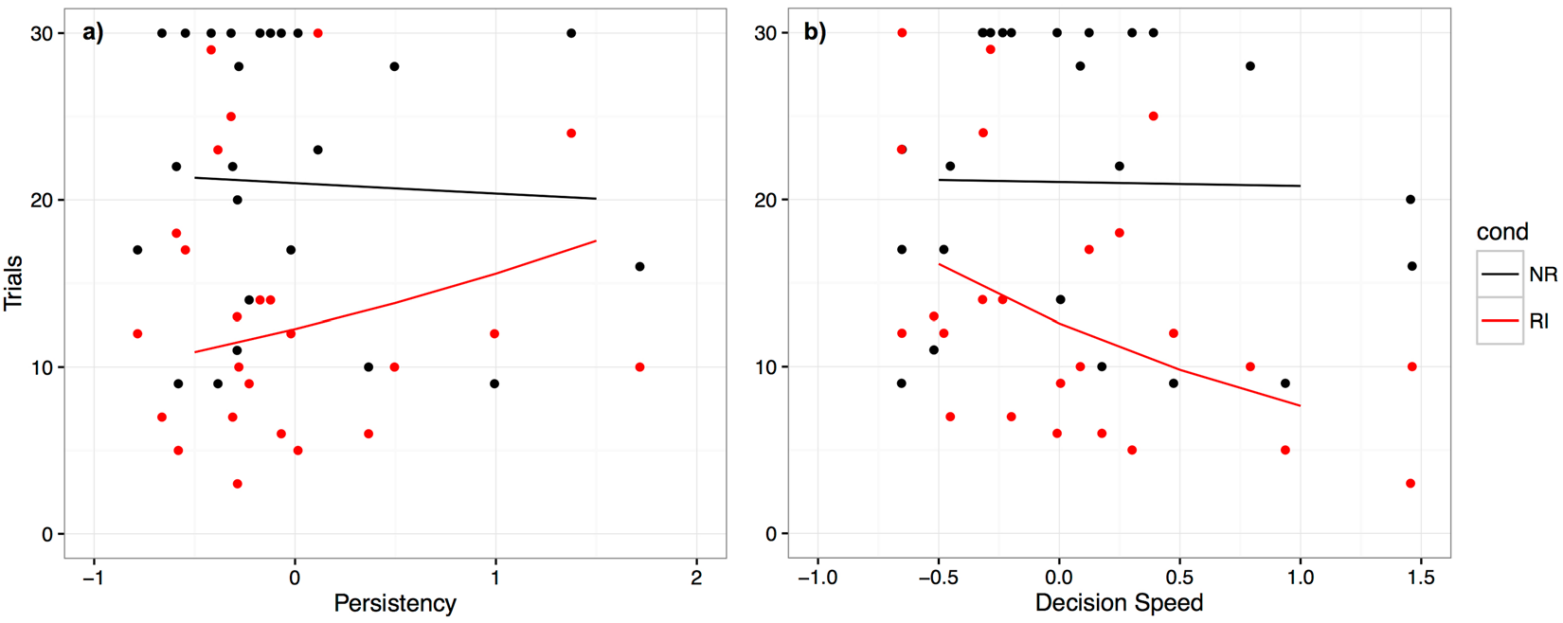
(**a**) Interaction between numbers of trials completed (= n. times paws given) per condition and the persistency component. A higher score on the persistency component is associated with a more persistent reaction in the inhibition tests. (**b**) Interaction between numbers of trials completed per condition and the decision speed component. A higher score on the decision speed component is associated with a slower reaction in the inhibition tests. Raw data (points) and predictions based on the selected model (colours: black = ET, red = FC, blue = QI, green = RI).

Accordingly, more persistent dogs gave their paw for longer than less persistent dogs (see Fig. 2a). Dogs exhibiting the same dis-advantageous behaviours repeatedly without giving up in the inhibition tests as assessed via the persistency component, reacted to reward inequity less strongly in comparison to the baseline condition (NR). Moreover, dogs with a slow decision speed stopped giving their paw earlier than faster dogs (see Fig. 2b). Hence dogs that take a longer time to make a decision in the inhibition tests also react more strongly to reward inequity than dogs that make faster decisions.

## Discussion

In accordance with our hypothesis stating that inhibitory control influences the expression of inequity aversion, we found that, overall, more compulsive dogs, i.e. those that consistently stuck to their initial choices regardless of subsequent feedback in the inhibition tests, were more motivated to participate in the task. Furthermore, when looking at the dogs’ behaviour in the different test conditions, we found that all conditions were affected by the inhibition component ‘decision speed’. Dogs that showed a slower decision speed in the inhibition tests also refused to give their paw earlier in both inequity conditions, but also in the contrast condition. In addition, the persistency component (i.e. dogs exhibiting the same behaviours repeatedly without giving up) accounted for individual variation in the reward inequity condition. Less persistent dogs showed a stronger aversive reaction to reward inequity than more persistent dogs. Finally, the impulsivity score obtained from the owner-reported questionnaire explained some of the variation observed in the reward inequity condition.

The overall motivation of dogs to give the paw in the inequity test was affected by the compulsivity component indicating that more compulsive dogs had fewer difficulties in performing the same action (i.e. give paw) repeatedly. Interestingly, this compulsivity component related to the overall willingness of dogs to perform the task but not to the variation within specific conditions. This influence of compulsivity on dogs’ motivation to participate in a task that involves a repetitive action might act as a limiting factor on the initial selection of animals that are participating in inequity studies. Dogs that did not comply with the precondition for assessing the dogs’ motivation (i.e. giving paw 15 times in a row), and also dogs that failed to reach criterion for being included in the final results (i.e. giving paw at least 20 times in the assessment condition) may be those with a lower level of compulsive and persistent behaviours. Similarly, Brosnan *et al*.^12^ found that the personality dimensions of openness, reactivity, agreeableness and dominance were negatively related to the overall number of refusals of chimpanzees in an inequity paradigm. Personality dimensions included behaviours like excitement, persistency, impulsivity but also inventiveness; indicating that chimpanzees scoring higher on these traits also completed more exchanges with the experimenter. In line with this, the ‘compulsive’ individuals might exhibit a higher working motivation in general. Nonetheless, it needs to be noted that we do not know how stable the inhibition measurements but also the inequity aversion measurements are over time. Despite keeping the duration between both tests to a minimum, test-retest reliability was not assessed in the current study. Future studies need to investigate how stable these behavioural traits are.

In order to understand the relationship between dogs’ inhibitory control abilities and their reaction to inequity we investigated the influence of certain inhibition components on the behaviour in each test condition in relation to the baseline conditions. One crucial component seems to be decision speed (see Table 2 for variables loading on this component), which influenced dogs’ behaviour in both inequity conditions, but also in the contrast condition. Accordingly, dogs that take their time to reach a decision, and hence do not act impulsively, seem to recognize the discrepancy between reward types offered to them (contrast condition) and/or offered to their partner (inequity conditions) more easily than dogs that just react very quickly and consequently might miss the difference between payoffs. This effect might be mediated by an increased attention in observing the interactions between partner dog and experimenter. Such a trade-off between accuracy and speed has been documented in humans, revealing that personality differences exist between ‘impulsive’ and ‘reflective’ subjects (e.g. ref.^29^). But also in non-human animals inter-individual variability in decision speed and accuracy have emerged, with individuals that make decisions very fast showing higher error rates than individuals that take more time to choose (see ref.^30,31^ for reviews). This might be due to the fact that fast-acting individuals do not recognize the important features of the given task, such as, in our study, the differences in reward quality.

In contrast to the decision speed component, an effect of persistency only emerged when using the asocial no-reward condition as baseline. Consequently, the less persistent dogs might get frustrated more easily in strongly unequal situations, whereas more persistent dogs try to perform the required action for longer, regardless of payoff. Similarly, Range *et al*.^11^ found that dogs’ motivation to give the paw in the non-rewarded asocial control condition, using the same inequity paradigm, was positively correlated with their motivation to manipulate a puzzle box. This could indicate that persistent behaviour plays a bigger role when the dogs are not being rewarded. Indeed, different aspects of inhibitory control are likely required when no reward is offered compared to test conditions in which a reward of lower quality is offered. In unrewarded conditions, dogs need to inhibit obeying the command, similar to an extinction paradigm but in unequal reward conditions dogs need to inhibit obeying the command but crucially by doing so they also refuse to take the offered reward albeit of lower quality than that of their partner. Consequently, situations in which no reward is given likely elicit more frustration with which the dogs need to cope. More persistent dogs might continue to give the paw with the hope to receive a reward at some point, due to better coping mechanisms (e.g. positive cognitive bias), while less persistent dogs simply give up. This persistency aspect might be of less importance in rewarded conditions because those are less frustrating; hence there might be no need for explicit coping strategies.

Interestingly, dogs’ sensitivity to reward inequity was also positively related to the impulsivity score obtained from the DIAS questionnaire^28^ indicating that more impulsive dogs were more sensitive to reward inequity, which is in contrast to our predictions. When investigating inhibitory control in dogs^26^, we found that only one sub-score (‘responsiveness’) obtained from the DIAS questionnaire was negatively correlated with the ‘decision speed’ component, but not with the other two inhibition components derived from the test battery. Accordingly, dogs that were rated more responsive and aware of their environment also made faster decisions in the inhibition test battery. Nonetheless, this general lack of correlations suggests that different aspects of inhibition are assessed with the questionnaire compared to the battery. Indeed, many questions refer to daily situations, in which the dog may be excited (e.g. repetitive behaviours when excited, behaviours exhibited in new situations), hence capturing the dogs’ ability to cope with arousal rather than their inhibitory control abilities. Consequently, those dogs that cannot cope well in taxing situations during daily life might be more sensitive/reactive and thus also react more strongly to inequity. Considering the nature of inequity aversion paradigms involving the distribution of unequal food rewards, tasks assessing inhibitory control in a food context might be more appropriate to assess the effect of inhibition on cognitive tasks than questionnaires assessing inhibitory control in daily situations, often outside of the food context.

Overall our results show that inhibitory control abilities can affect an individual’s reaction to inequity. This however, does not necessarily mean that the more impulsive individuals are less sensitive to unequal treatment; rather, they may have greater difficulties in *showing* this sensitivity in the current setup where aversion is measured in terms of refusal, or abstaining from action. If an individual cannot refrain from obeying the given command and/or from taking the offered reward, it cannot show an aversion to the unequal treatment, even though it might very well recognize the inequity being perpetrated. It therefore becomes important to devise additional, simpler ways to measure an individual’s sensitivity to inequity. One such method was used in Brucks *et al*.^27^, in which the dogs’ social behaviour towards the better-rewarded partner dog and the experimenter who caused the inequity was observed following the various test conditions. There we found that dogs, which did not show a primate-like reaction to quality inequity, still avoided the experimenter but also the conspecific partner after the quality inequity condition. In addition, the unequally treated dogs showed reduced co-feeding behaviour with their better-rewarded partner following the inequity test^27^. This indicated that dogs were likely not able to show their aversion to inequity in the test context due to various reasons (e.g. lack of inhibitory control), even though they noticed the inequity. Consequently, the sensitivity to unequal treatment can be observed also with tasks that do not require high levels of inhibitory control (e.g. sharing resources, social behaviour following unequal treatment). Since this incorporation of additional measures has so far only been done in dogs, it could be an interesting avenue for future research and might reveal that inequity aversion is present but hindered by a lack of inhibition control, also in other species.

## Methods

Twenty-four dogs (10 M/14 F) that participated in the initial inequity study^27^ (mean age ± SE: 4.9 ± 0.48 yrs.) were subsequently tested in an inhibitory control battery (mean age ± SE: 6.7 ± 0.48 yrs., see Supplementary Material Table S1). Even though inhibitory control seems to be a rather stable trait (e.g. dogs^32^, humans^33^) we tried to keep the duration between the inequity test and the inhibition test battery to a minimum (1.8 ± 0.1 years). All procedures were discussed and approved by the ethics commission of the University of Veterinary Medicine Vienna in accordance with Good Scientific Practice guidelines and national legislation (Approval numbers: 10/12/97/2013, 08/08/97/2013).

### Inequity Test

**Figure 3:**
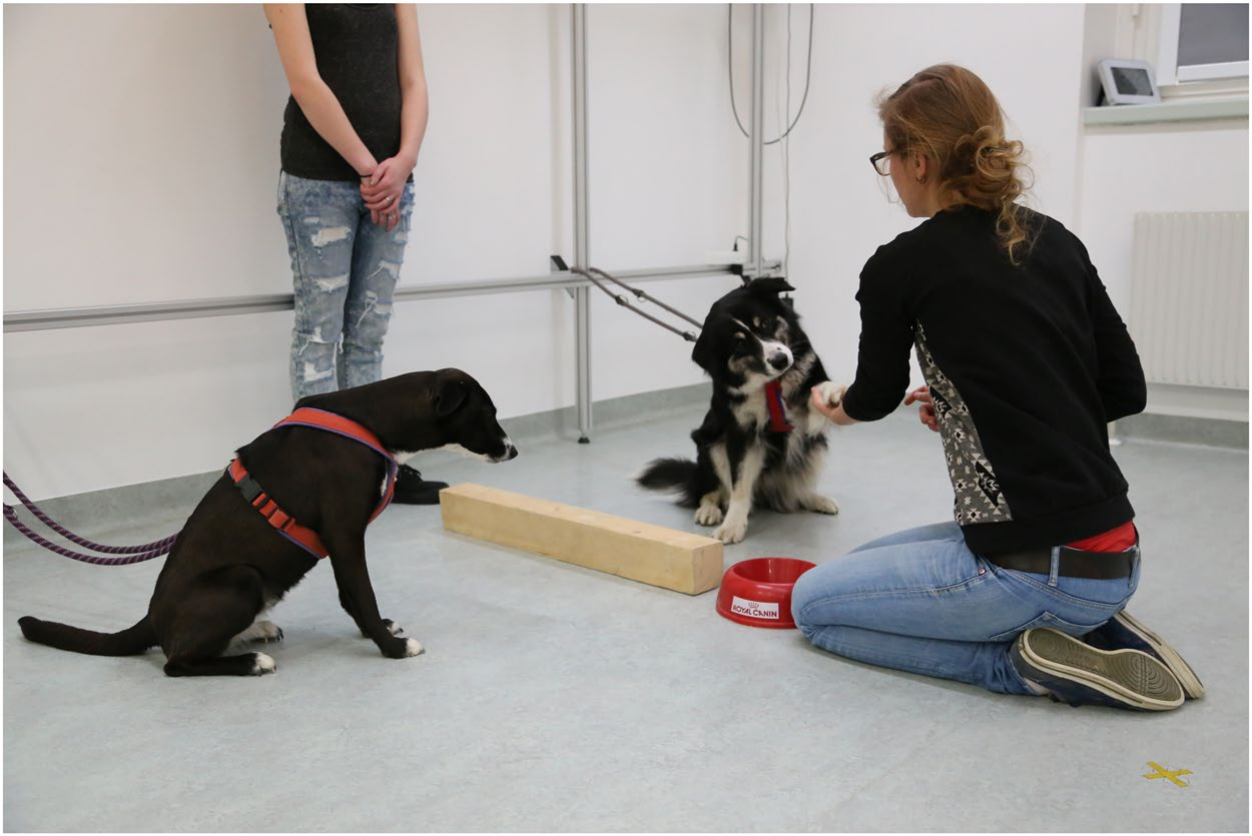
Setup for inequity test. The owner is standing behind the dogs, while the experimenter is kneeling in front of the dogs with a bowl containing both reward types in-between her legs.

Dogs living together in pairs in the same household for at least one year were tested. As in previous studies (e.g. ref.^5,6,34^), the pre-condition for participating in this paw-giving paradigm was that each dog within a dyad would give its paw for 15 times in a row, with and without being rewarded (i.e. 5x rewarded, 5x not rewarded, 5x rewarded). During the test, the dogs were sitting next to each other attached by leashes to the wall. A wooden block was placed on the ground in-between the dogs as a ‘physical’ barrier to prevent dogs from changing sides. The owner was standing behind the dogs facing straight ahead without interacting with them. An experimenter was kneeling in front and facing the dogs at a distance of 50 cm. A food bowl containing an equal amount of high-value rewards (HVR) and low-value rewards (LVR) was in-between the experimenter’s legs (see Fig. 3). Note that the reward types used for each dog dyad were determined before the beginning of the test by conducting a food preference test, in which the dogs were repeatedly presented with both reward types in a two-way choice setup (see ref.^27^ for details on procedure). Only reward types, which both dogs within a dyad clearly distinguished and showed the same preference for, were used in the inequity test. The dogs needed to be in a sitting position before the experimenter asked them to give their paw, by holding her hand outstretched in front of the dog and by giving the verbal command ‘paw’ (see ref.^27^ for detailed test procedure; Fig. 3). As soon as the dog gave its paw, the experimenter reached into the food bowl and handed the dog a reward.

Prior to testing, the dogs were assigned the roles of subject and partner and those roles were switched only after the first dog had completed all conditions. The partner dog was always asked for the paw first, then the same command was repeated to the subject. When the dogs gave the paw, they received either 1) the same reward (equity condition), 2) rewards of different quality (quality inequity condition), 3) the same reward but showing the more preferred reward beforehand (food control/frustration condition) or 4) one dog received a reward, while the other dog did not (reward inequity condition) (see Table 3). In addition, the dogs were tested in two non-social conditions, in which they were either rewarded (assessment control) or not rewarded (no-reward control). The assessment condition aimed to assess if dogs were motivated enough to give their paw 30 times in a row, while the no-reward control served as an asocial control for the reward inequity condition testing whether the animals would indeed work for 30 trials even if they received no reward. Each test condition lasted until either one dog refused to give its paw after 10 command repetitions, or until 30 trials were completed. Two test conditions were conducted per test day with a 10-minute break between conditions.

**Table 3:** Test conditions for inequity task (adapted from ref.^27^).

Condition	Subject	Partner
*Social Conditions*		
Equity (ET)	LVR	LVR
Quality Inequity (QI)	LVR	HVR
Reward Inequity (RI)	No reward	HVR
Food Control (FC)	HVR moved, LVR given	HVR moved, LVR given
*Asocial Conditions*		
Assessment Control (AC)	LVR	−*
No Reward Control (NR)	No reward	−*

*In the asocial conditions, the LVR bowl was moved into the empty enclosure first before the subject’s trial started, in order to rule out that dogs just react to the movement of the food but not the outcome of the partner (see also ref.^6^).

### Inhibitory Control Test Battery

The test battery consisted of two motor inhibition tasks (box test and middle cup test), one cognitive inhibition task (reversal learning test), a delay of gratification task and one newly- designed task with both motor and cognitive inhibition aspects (buzzer test). Dogs were tested only in one test per day and the order of tests was randomized (see Supplementary Material and^26^ for detailed descriptions of procedures).

**Figure 4:**
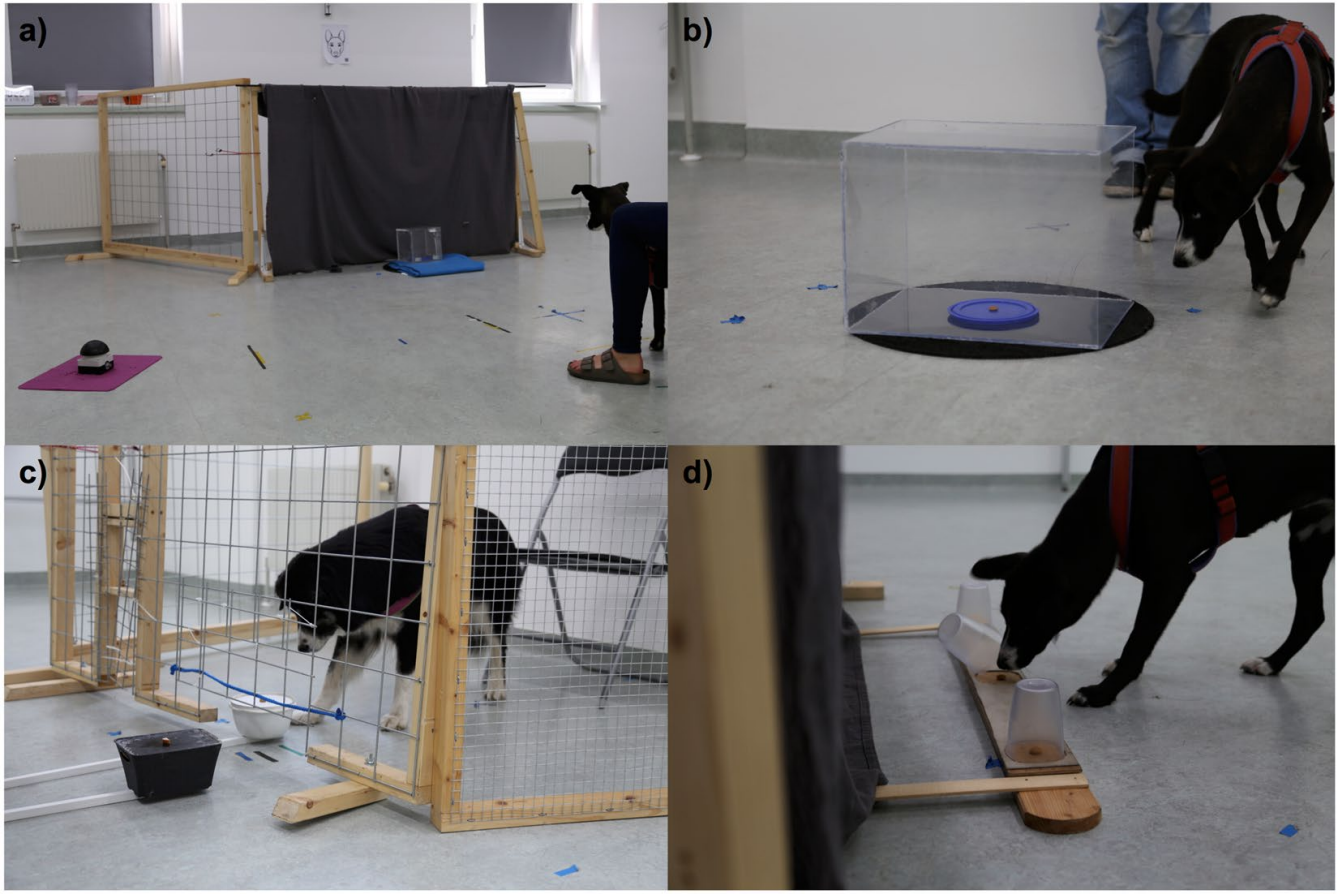
(**a**) Buzzer test: the dog needs to press the buzzer to open the transparent box containing a reward. (**b**) Box test: the dog is searching for the open side of the transparent cube in order to get the reward. (**c**) Delay of gratification test: the dog is waiting for the piece of sausage and refraining from eating the already accessible piece of dry food. (**d**) Middle cup test: the dog knocks over the middle cup in the control condition, in which adjacent cups are baited.

In the *buzzer test*, dogs were trained to press a buzzer to open a Plexiglas box, which contained a piece of sausage (see Fig. 4a). Since dogs could see the reward visibly being placed inside the transparent box at the beginning of each trial, they had to refrain from manipulating and staying close to the box but instead turn away and press the buzzer placed 2 m away from the box. Five test trials were conducted and if a dog did not press the buzzer within 60 seconds, the trial was scored as a failure and the next trial started (see Table 4).

**Table 4:** Overview of variables coded for each inhibition test and the components derived from a principle component analysis (see ref.^26^).

Inhibition Test	Aspect of inhibition	Variables	Component
*Buzzer*	Manipulating/staying close to reward	Number of successful trials	**Persistency**
		Duration close to the box	
		Duration manipulating the box	
		Latency to press the buzzer	
* Box*	Reach for reward directly	Number of successful trials	**Persistency**
		Number of surface touches	
		Duration close to cube (centre, deep)	Persistency/Decision speed*
		Latency to retrieve reward (centre, deep)	
*Delay of Gratification*	Eat immediately accessible reward	Maximum delay tolerated	**Persistency**
*Middle Cup*	Knock over adjacent cups	Number of successful trials (control, experimental)	**Compulsivity**
		Latency to choice (control, experimental)	**Decision speed**
		Duration close to cups	
*Reversal Learning*	Choose previously rewarded object	Number of successful trials (acquisition, reversal)	**Compulsivity**
		Latency to choice (acquisition, reversal)	**Decision speed**
		Duration close to objects (acquisition, reversal)	

*These variables had cross-loadings on both components.

In the *box test*, dogs were confronted with a big Plexiglas cube, which was open only on one side. The experimenter showed the dog a piece of sausage on a plastic lid and placed the baited lid inside the cube. The dog was released and allowed to retrieve the reward from the transparent cube (see Fig. 4b). Dogs had to inhibit reaching for the reward from their closest side but instead look for the open side of the cube and get the reward from there (see Table 4).

In the *delay of gratification test*, dogs were given the choice between an immediately available low-quality reward and a delayed high quality reward. This high quality reward however, was only made accessible if the dog refrained from eating the low quality reward for the established length of time (ref.^35^; see Fig. 4c). Inhibitory control was measured in terms of the amount of time (delay) the dogs could withstand eating the initially offered (lower quality) reward (see Table 4).

In the *middle cup test*, dogs were presented with an alignment of three transparent cups. The dogs witnessed how two pieces of dry food were placed under either adjacent (see Fig. 4d) or non-adjacent cups and were then allowed to choose two cups (the third was removed prior to their getting to it). Hence when non-adjacent cups were baited, the dogs needed to inhibit choosing adjacent cups and go around the empty middle cup without knocking it over (see Table 4).

In the *reversal learning test*, dogs were first trained to discriminate between two objects, associating one of them with a reward. Once dogs had learned this association, reward contingencies were reversed. Consequently, the dogs needed to inhibit choosing the previously rewarded object to learn the new association (see Table 4).

### Impulsivity Questionnaire (DIAS)

In addition to the inhibition test battery, dog owners were asked to fill in a questionnaire about their dogs’ impulsivity in daily situations. This questionnaire has previously been validated (ref.^28^; dog impulsivity assessment score = DIAS), and contains 18 questions, which are rated on a 5-point Likert scale. One overall impulsivity score was calculated from the questionnaire (see ref.^28^ for details).

### Analyses

The data from the inequity test was obtained from Brucks *et al*.^27^ and re-analysed including only the dogs that participated also in the inhibition tests, in order to validate that the inequity test elicited inequity aversion also in this subset of dogs (see Supplementary Material for results). The inhibition data was taken from Brucks *et al*.^26^. As measures of inhibition, we took the factors obtained from a principle component analysis (i.e. persistency, compulsivity and decision speed; see Table 4 for details on factor composition and^26^ for details on the analysis). We ran generalized linear mixed models (GLMM) with a Poisson distribution and log-link error function (R^36^, package ‘lme4′ ^37^). The number of times a paw was given on command in the inequity test was used as response variable and the dog identity as random effect. As fixed effects the scores of each inhibition component (persistency, compulsivity, decision speed), and the impulsivity score obtained from the DIAS questionnaire were entered. In order to investigate whether these inhibition components could serve as an explanation for the observed variation in paw-giving behaviour across test conditions, we analysed the data in three models. First, to assess whether dogs’ inhibition abilities alone predict how often they would give the paw independent of the test condition, we ran one model testing for the main effects of the inhibition measurements on the overall number of paws given across all test conditions (*Model 1: response variable: number of paws given; predictors: inhibition components*, *DIAS score*). Subsequently, to investigate if inhibitory control plays a differential role in the different test conditions, we assessed the interaction between inhibition measures and the paw-giving behaviour per test condition in relation to a baseline condition. The equity condition (ET), in which both dogs receive the same reward, served as baseline condition for the dyadic test conditions (i.e. food control (FC), quality inequity (QI), reward inequity (RI)). Whereas the asocial no-reward condition (NR) served as an additional baseline for the reward inequity condition in controlling for the effect of the partner dog’s presence in the unrewarded conditions. We ran two separate models: The first model explored the interaction between paw-giving behaviour in the food control, quality inequity, and reward inequity condition and the components derived from the inhibitory control battery compared to the equity condition (*Model 2: response variable: number of paws given; predictors: condition* (*ET*, *FC*, *QI*, *RI*), *inhibition components*, *DIAS score*). The second model assessed the interaction between paw-giving in the reward inequity condition and the inhibition measures compared to the no-reward condition (*Model 3: response variable: number of paws given; predictors: condition* (*NR*, *RI*), *inhibition components*, *DIAS score*). We used the Akaike information criterion (AIC) to reduce the full model in order to find the best fit.

## Electronic supplementary material

Supplementary Material
