# Supplementary material for: Dogs’ reaction to inequity is affected by inhibitory control
Source: Sci Rep. 2017 Nov 17;7:15802. doi: 10.1038/s41598-017-16087-w (PMC5694007; doi:10.1038/s41598-017-16087-w)
Supplement: Supplementary file 1 — Supplementary Material [file 41598_2017_16087_MOESM1_ESM.pdf]

Dogs' reaction to inequity is affected by inhibitory control

Désirée Brucks*, Friederike Range, & Sarah Marshall-Pescini

Comparative Cognition Unit, Messerli Research Institute, University of Veterinary Medicine Vienna, Medical University Vienna, University of Vienna, Austria

* Correspondence: desiree.brucks@me.com

Supplementary Material

Table S1. Individual characteristics and questionnaire (DIAS) score of dogs participating in inequity aversion (IA) and inhibitory control (IC) study.

Dyad	Name	Age (yrs.) IA	Age (yrs.) IC	Sex	Breed	DIAS score
1	Achuk	8.3	9.8	F	Chesapeake Bay Retriever	0.51
1	Elrond[x]	4.6	6.1	M	Chesapeake Bay Retriever	0.71
2	Aiko	2.4	4.2	M	Australian Shepherd	0.62
2	Emely	1.7	3.4	F	Bernese Mountain Dog	0.68
3	Baja	1.1	3.7	F	Australian Shepherd	0.56
3	Daimony	6.7	8.3	F	Australian Shepherd	0.50
4	Bella	6.5	8.2	F	Bernese Mountain Dog	0.41
4	Brandy	3.0	4.8	F	Bernese Mountain Dog	0.64
5	Chasie	4.8	6.4	F	Border Collie	0.42
5	Gatsby	3.5	5.2	M	Border Collie	0.51
6	Cole	6.4	7.9	M	Border Collie	0.49
6	Esprit	3.7	5.2	F	Border Collie	0.43
7	Emily	5.7	7.3	F	Border Collie	0.44
7	Ziva	2.1	4.8	F	Border Collie	0.69
8	Luke	8.0	9.8	M	Border Collie	0.52
8	Quismo	6.1	7.8	M	Border Collie	0.51
9	Luna	1.2	2.8	F	Siberian Husky	0.49
9	Talie	2.8	4.3	M	Siberian Husky	0.54
10	Mago	9.1	10.9	M	Golden Retriever	0.36
10	Tika	6.1	8.6	F	Husky-Mix	0.41
11	Pippilotta	7.1	9.7	F	Irish Terrier	0.49
11	Poquita	4.1	6.5	F	Galgo Espagñol	0.43
12	Sokrates	7.9	9.5	M	Bardino-Mix	0.38
12	Ultimo	4.2	6.0	M	Border Collie	0.50

[x] *dog completed only four inhibition tests*

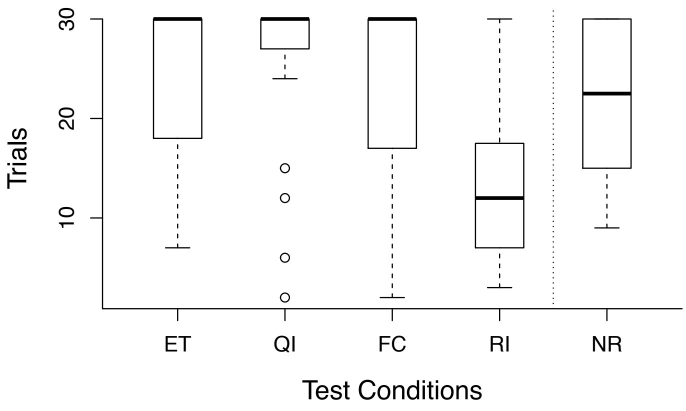

Figure S1. Number of paws given across test conditions in the inequity test (N = 24 dogs). Circles show outliers, black bars indicate median values, whiskers display upper and lower hinge, and boxes show the interquartile range.

Detailed Methods Inhibition Tests

Box Task

A rectangular-shaped Plexiglas box (40x30x30 cm), which was open on one of the smaller sides, was utilized for this test. Pieces of sausage (2cm) were used as rewards and placed on a blue plastic lid inside of the box. The dog was released 2m away from the box during each trial.

In the *training phase*, an opaque box was used and the dog witnessed how the experimenter placed the reward inside of the box. Six trials were conducted while alternating the open side of the box in-between trials (i.e. left, right, back).

Following the training phase, the *test phase* began, in which a transparent box was presented. The dog was no longer allowed to witness the baiting process (a curtain was placed in-between dog and box while the experimenter placed the reward inside), and the dog was released as before to retrieve the reward. A trial stopped when the dog retrieved the reward or when 30s had passed. As in the training, six trials were conducted while alternating the open side of the box. In addition, the rewards were either placed at a centre position (15 cm inside) or deep position (30cm inside).

We coded the frequency of errors (= surface touches with either nose or paw), the latency to success (= time from release until nose enters box, max. 30s), and the time spent in proximity to the box (= within 1m radius).

Buzzer Task

A Plexiglas box (25x25x25 cm) that could be opened via a latch was used in this test. The box was positioned in front of a curtain and could be opened via a string from behind a curtain. Furthermore, a buzzer (Eaton ®) was placed next to the box (50 cm either to left or right) and in an initial training phase the dogs were taught to associate pressing the buzzer with the box opening (see [1] for details). The box was covered with fabric so that the dogs could not see the hidden reward inside during the training phase.

After the experimenter visibly baited the box, the dog was released from a position 2m away from the box. As soon as the dog pressed the buzzer, a second experimenter opened the box from behind the curtain and the dog could retrieve the reward. If a dog did not press the buzzer within 30s, the experimenter helped in pointing towards the buzzer. When a dog pressed the buzzer without the experimenter's help in 7 consecutive trials, the training phase was terminated and the test phase started.

In the test phase, the buzzer was position 2m away from the box (either to left or right, but same side as in training) and the cover was removed from the box so that it was transparent. As before, the experimenter visibly baited the box and during the first trial touched the buzzer before walking to a location behind the dog. The dog was released and as soon as the dog pressed the buzzer the box was opened. If a dog did not press the buzzer within 60s, the trial was terminated and the next trial started after a 30s break. In total five trials were conducted.

We coded the following variables: proximity to box (= within 1m radius), latency to press buzzer (= time from release to press buzzer, max. 60s), duration of manipulation (= scratching box with paw or pushing with nose), number of successful trials (= buzzer pressed within 60s).

Delay of Gratification Task

An enclosure made out of three separate fences with mesh was arranged (2 m^2). In addition,

two plastic bowls (= black and rectangular (15x7cm), white and round (15cm diameter)) attached to sticks were used for this test. The bowls could be moved inside and out of the enclosure through a hole in the front fence. In front of the fences a wooden frame with a curtain was positioned, in order to allow the experimenter to manipulate the bowls invisible to the dog.

Prior to testing, food preference tests were conducted to establish two reward types of different quality to the dogs (i.e. LVR (low value reward; e.g. carrots) and HVR (= high value reward; e.g. sausage) (see [2] for details of this procedure). Moreover, the dogs were familiarized with the bowls and the containing reward types in association trials, in which the dogs were repeatedly presented with the rewards on the bowls (reward type and bowl colour counterbalanced across dogs), and were allowed to choose one bowl. Twelve association trials were conducted with alternating the sides of the rewards (see [2] for detailed description).

Following this, the training phase began. Dog and owner entered the enclosure and the owner sat down on a chair inside of the enclosure while keeping the dog on a leash. During each trial, the dog was presented with an immediate option (= LVR) and a delayed option (=HVR), however, the delayed option was only made accessible when the immediate option was not consumed by the dog. Two different types of trials were conducted in the training phase: *demonstration trials,* in which the owner restraint the dog from eating the immediately available reward and only released him/her when the delayed reward was accessible; and *test trials,* in which the owner did not restrain the dog and the dog was free to choose between the immediate and the delayed reward. Five trials of each trial type (demonstration and test) were conducted per session. The delay between reward types was set to 1s in the training phase, and if a dog waited for at least three test trials in the training phase the test phase began. If a dog did not reach this criterion within four sessions, the delay of gratification test was terminated for this dog, and a maximum delay of 0s was noted in this case. No more than three sessions were conducted per test day.

In the test phase, the owner left the enclosure and the dog was free to choose between reward options. At the beginning of each session four demonstration trials were conducted, in which the owner restraint the dog from choosing the immediate option (as before in the training phase). These demonstration trials were included in order to allow the dog to familiarize with the upcoming delay duration. Ten test trials (= owner invisible) were conducted per session and if a dog waited for the delayed option in at least three test trials during one session, he/she commenced to the next delay stage in the following test session. If a dog did not pass this criterion within four sessions, the test was terminated. The delay was slowly increased between sessions, starting with 2s, then 5, 10, and up to 20s. The main variable of the test was the maximum delay tolerated by each dog.

Middle Cup Task

Three transparent cups of 11cm height were aligned on a wooden board (30cm in-between cups) in front of a curtain. The dog was released from a position 2m away from the board, while the experimenter was manipulating the cups from behind a curtain.

Dogs were familiarized with the task during two warm-up trials, in which they learnt to knock over the cups in order to gain access to the rewards. In the test phase, the experimenter visibly baited two of the three cups before the dog could make two choices. In *experimental trials*, two non-adjacent cups were baited (i.e. left and right), while in *control trials* two adjacent cups were baited (e.g. left and middle; right and middle). Twenty trials were conducted, while randomizing the occurrence of the trial types (10 experimental and 10 control trials).

We coded the number of successful choices (= knocking over only baited cups) in both trial types, the latency to make both choices (= release until second cup was chosen), as well as the time spent in vicinity to the cups (= within 1m radius).

Reversal Learning Task

Dogs had to discriminate between two different objects (orange rectangular felt object and blue round plastic object) in this test. The dogs were released from a position 2m away from the objects, which were manipulated by an experimenter from behind a curtain.

In the *acquisition phase* dogs learnt to discriminate between the objects, while the choice of only one object was rewarded (rewarded object counterbalanced between dogs). The acquisition phase started with four warm-up trials in which only the rewarded object was present and as soon as the dog approached this object a click from a secondary reinforcer was emitted and the object was lifted so that the dog could retrieve the reward from under it. Following the warm-up trials both objects were simultaneously presented while only the rewarded object was baited. If the dog approached the rewarded object, a click sound was emitted and the experimenter lifted the object. If the dog chose the negative object, no sound was emitted and the empty object was lifted. After each trial, the dog was called back to the starting position and the next trial started. The location of rewards was semi-randomized (not more than two times on the same side in a row). Twelve trials were conducted per session and if a dog reached the criterion of choosing the rewarded object in at least 9 trials (binomial: $p<0.02$) the acquisition phase was terminated. If the criterion was not met, another session was conducted, but not more than two sessions per test day. A maximum of four acquisition sessions were conducted in total and all dogs learnt to discriminate between the objects within this time.

In the *reversal phase,* the object contingencies were reversed and the rewarded object from the acquisition phase was not rewarded anymore, instead the negative object was rewarded now. The reversal phase started with four warm-up trials, as described above, and twelve trials were conducted following the procedure of the acquisition phase, except for not omitting a click sound during correct choices. No criterion was set in the reversal phase and after one session the test was terminated.

We coded the number of correct choices in reversal and last acquisition phase, the latency to make a choice (= release until touched object), and the time spent in vicinity to the objects (= within 1m radius).

Results Inequity Test

While dogs gave their paw equally often in the quality inequity and food control condition compared to the equity condition (Wilcoxon Test: QI-ET: $T = 31$, $N = 24$, $p = 0.893$ after Bonferroni; FC-ET: $T = 39$, $N = 24$, $p = 0.623$ after Bonferroni; see Fig. S1), they refused to give their paw earlier in the reward inequity condition (RI) compared to the equity condition (ET; Wilcoxon Test: $T = 263$, $N = 24$, $p < 0.001$ after Bonferroni; Fig. S1). Moreover, dogs stopped giving they paw earlier if they witnessed their partner receiving a reward whilst they were not rewarded (RI) compared to the asocial condition (NR), in which they were likewise not rewarded, but importantly no partner was present (Wilcoxon Test: $T = 267$, $N = 24$, $p < 0.001$ after Bonferroni; Fig. S1).

REFERNCES

1. Brucks, D., Marshall-Pescini, S., Wallis. L.J., Huber, L., & Range, F. Measures of dogs' inhibitory control abilities do not correlate across tasks. *Front. Psychol.* **8**, 849 (2017).
2. Brucks, D., Soliani, M., Range, F., & Marshall-Pescini, S. Reward type and behavioural patterns affect dogs' success in a delay of gratification task. *Sci. Rep.* **7**, 42459 (2017).
